# Unified segmentation based correction of R1 brain maps for RF transmit field inhomogeneities (UNICORT)

**DOI:** 10.1016/j.neuroimage.2010.10.023

**Published:** 2011-02-01

**Authors:** Nikolaus Weiskopf, Antoine Lutti, Gunther Helms, Marianne Novak, John Ashburner, Chloe Hutton

**Affiliations:** a Wellcome Trust Centre for Neuroimaging, UCL Institute of Neurology, University College London, London, WC1N 3BG, UK; b MR-Research in Neurology and Psychiatry, University Medical Center, Göttingen, Germany

**Keywords:** B1 inhomogeneities, RF transmit field inhomogeneities, Bias correction, T1 mapping, Quantitative MRI, B1^+^

## Abstract

Quantitative mapping of the longitudinal relaxation rate (R1 = 1/T1) in the human brain enables the investigation of tissue microstructure and macroscopic morphology which are becoming increasingly important for clinical and neuroimaging applications. R1 maps are now commonly estimated from two fast high-resolution 3D FLASH acquisitions with variable excitation flip angles, because this approach is fast and does not rely on special acquisition techniques. However, these R1 maps need to be corrected for bias due to RF transmit field (B1^+^) inhomogeneities, requiring additional B1^+^ mapping which is usually time consuming and difficult to implement. We propose a technique that simultaneously estimates the B1^+^ inhomogeneities and R1 values from the uncorrected R1 maps in the human brain without need for B1^+^ mapping. It employs a probabilistic framework for unified segmentation based correction of R1 maps for B1^+^ inhomogeneities (UNICORT). The framework incorporates a physically informed generative model of smooth B1^+^ inhomogeneities and their multiplicative effect on R1 estimates. Extensive cross-validation with the established standard using measured B1^+^ maps shows that UNICORT yields accurate B1^+^ and R1 maps with a mean deviation from the standard of less than 4.3% and 5%, respectively. The results of different groups of subjects with a wide age range and different levels of atypical brain anatomy further suggest that the method is robust and generalizes well to wider populations. UNICORT is easy to apply, as it is computationally efficient and its basic framework is implemented as part of the tissue segmentation in SPM8.

## Research Highlights

►UNICORT estimates RF transmit inhomogeneities and corrected R1 values from data. ►Performance similar to widely used corrections without need for special sequences. ►Allows for high-resolution R1 mapping with standard tools. ►R1 mapping enables the investigation of tissue microstructure.

## Introduction

Quantitative mapping of the longitudinal relaxation provides absolute values of the longitudinal relaxation time T1 and longitudinal relaxation rate R1 (= 1/T1), which makes the results system independent and separates different sources of contrast (Tofts, 2003). Thus, quantitative magnetic resonance imaging (MRI) improves the comparability and interpretability of results in comparison to the widely used T1-weighted (T1w) MRI (Ashburner et al., 2003). The latter exhibits a contrast depending primarily, but not solely on T1 relaxation and does not directly provide absolute values of the T1 time. Quantitative R1 mapping has been successfully used to study the morphology and the microstructure of brain tissue (Tofts, 2003).

A method based on acquisitions of 3D FLASH with variable excitation flip angles (VFA) is popular due to its speed, high signal-to-noise ratio (SNR) and therefore its ability to provide R1 maps at high resolution with high precision (Deoni et al., 2003; Deoni, 2007; Helms et al., 2008a). However, at high static field strengths (> 1.5 T), the radio-frequency (RF) transmit field (here, also called B1^+^ field) is significantly distorted leading to systematic deviations of the local excitation flip angle across the brain (Lutti et al., 2010a) and hence inaccurate R1 values.

When the local flip angle (or B1^+^ field) is known, the R1 values can be corrected using post-processing procedures (Helms et al., 2008a). For this the local flip angle needs to be measured by dedicated B1^+^ mapping MR sequences (Lutti et al., 2010a). This requires extra scan time and — more importantly — fast whole-head B1^+^ mapping sequences which are not readily available on current clinical scanners. Careful post-processing steps must also be taken in order to achieve good results (Lutti et al., 2010a; Preibisch and Deichmann, 2009).

We have developed and validated a method for correction of RF transmit inhomogeneities in R1 maps that does not require the acquisition of B1^+^ maps. The method primarily uses the probabilistic framework for simultaneous image segmentation, registration and correction of multiplicative bias recently implemented in SPM8 for unified segmentation (Ashburner and Friston, 2005) — named “New Segment” toolbox (see Appendix A for details). This segmentation method can be used to estimate and remove the signal bias from any type of data suffering from a multiplicative bias where intensity distributions from different tissue classes are sufficiently separated.

Our unified segmentation based correction of R1 maps for RF transmit inhomogeneities (UNICORT) exploits the fact that the local flip angle varies smoothly across the human brain (Lutti et al., 2010a) and simply scales the local R1 value (Helms et al., 2008a). Thus, the measured R1 maps with RF transmit inhomogeneities can be described by the theoretically accurate R1 maps multiplied by a smooth bias field. Furthermore, the R1 values in gray matter (GM), white matter (WM) and cerebrospinal fluid (CSF) can be described by a mixture of Gaussian distributions across the brain (Oros-Peusquens et al., 2008). We validated the UNICORT correction in two different groups of subjects and two additional subjects with atypical brain anatomy by comparing estimated B1^+^ maps with those measured using an established B1^+^ mapping method and the corresponding R1 maps after correction with the estimated and measured B1^+^ maps.

## Methods

### Model description

This section describes the main physical properties of the bias field in R1 maps and shows how unified segmentation can be used to estimate the RF transmit inhomogeneities. Detailed information about the unified segmentation itself is given in Appendix A.

In the variable flip angle (VFA) R1/T1 mapping method, the local R1 value is estimated from at least two FLASH images acquired with different nominal flip angles (α_l_ and α_2_), which are typically a proton density weighted (PDw) and T1w image. For the two images, the signal amplitudes at a voxel are denoted by S_1_ and S_2_, respectively. A simple algebraic estimate of the apparent R1_app_ is based on the rational approximation of the Ernst equation (Helms et al., 2008a,b; Dathe and Helms, 2010):

(1) $$R1_{\text{app}}=\frac{1}{2}\;\frac{S_{2}\alpha_{2}/TR_{2}-S_{1}\alpha_{1}/TR_{1}}{S_{1}/\alpha_{1}-S_{2}/\alpha_{2}}$$

In Eq. (1), the sensitivity profiles of the receive coils cancel by division, but deviations of the local flip angles (α_local_) from their assumed nominal values (α, as entered on the scanner console) impose errors on R1_app_. At high static magnetic fields α_local_ may deviate significantly (by up to 30% at 3 T) from α due to the relatively short RF wavelength in comparison to the dimension of the head (Lutti et al., 2010a). Therefore, the calculation of R1 needs to be based on the actual local flip angle instead of the nominal flip angle. Let ψ = α_local_/α be the ratio of the local over the nominal flip angle due to the local B1^+^ field inhomogeneity, and S_n_ = S(ψ α_n_), be the signal of the two images *n* = 1, 2. Then the R1 value corrected for RF transmit field inhomogeneities can be estimated from R1_app_ (Helms et al., 2008a):

(2) $$R1=R1_{\text{app}}\psi^{2}$$

Due to the multiplicative nature of the bias, R1 and ψ can also be directly estimated from the R1_app_ maps using a probabilistic framework for segmentation (Ashburner and Friston, 2005). Briefly, the unified segmentation combines image registration, tissue classification, and bias correction in a single generative model and optimizes its log-likelihood objective function. We used the extended version of the segmentation and bias correction with an improved registration model, an extended set of tissue probability maps and a different treatment of mixing proportions, as implemented in the “New Segment” toolbox in SPM8 (for details, see Appendix A).

The model assumes that the brain image can be partitioned into GM, WM, CSF and non-brain tissue classes whose signal distribution can be described by a mixture of Gaussian distributions. This was empirically demonstrated for T1 at various field strengths (Oros-Peusquens et al., 2008), although the actual T1 value slightly varies across the specific tissue compartment (Ostergaard et al., 1998; Steen et al., 2000).

Further, the unified segmentation requires that the modelled bias (η) in signal amplitude is multiplicative and smoothly varying across space. It is evident from Eq. (2) and studies on RF transmit field inhomogeneities (Lutti et al., 2010a; Sled and Pike, 1998) that R1 maps fulfill this criterion. Moreover, it is assumed that the mean flip angle across the head equals the nominal flip angle entered on the scanner console. This is a reasonable assumption because clinical MR scanners run adjustment procedures to calibrate the RF transmit amplitude before image acquisition. In the generative model, the bias field is modelled by the exponential of a linear combination of cosine basis functions. User-defined smoothness (FWHM) and regularization parameters (κ) are set, such that the model avoids over-fitting.

UNICORT estimates the coefficients that parameterize the smooth bias field η, allowing corrected R1_UNICORT_ maps to be computed:

(3) $$R1_{\text{UNICORT}}=R1_{\text{app}}/\eta$$

By comparison of Eqs. (2) and (3), UNICORT also intrinsically yields an estimate of the RF transmit field (B1^+^) inhomogeneities by:

(4) $$\psi_{\text{UNICORT}}{}^{2}=1/\eta$$

Note that UNICORT could alternatively be applied to T1 maps instead of R1 maps. However, the performance of the correction is improved when applied to R1 maps (compare results of this study with Weiskopf et al. (2010)). Using R1 maps for the bias field estimation leads to a smaller influence of voxels with a high CSF content and hence small R1 values, and a larger influence of the rather homogenous WM voxels with high R1 values. Since the estimation of the long T1 values of CSF are less reliable than those of GM and WM, using R1 maps reduces the error in the bias field estimation.

### Participants

UNICORT was tested on two different groups of subjects and two additional subjects who were scanned as part of a study of Huntington disease and Parkinson's disease.

The first group consisted of 8 healthy volunteers (*healthy volunteer group*; 4 females) with normal brain anatomy spanning the adult age range from 23 to 64 years (mean ± standard deviation = 46 ± 17 years) selected from an original dataset of 10 volunteers (one subject was excluded due to motion artifacts, another showed atypical brain anatomy). The regularization and smoothness of the modelled bias field used in UNICORT was optimized on this group of 8 healthy volunteers. The second group consisted of 8 presymptomatic Huntington disease gene mutation carriers (*PSC group*; age = 47 ± 8 years, age range = 38–65 years; 6 females). This group was included in the study to assess how well UNICORT generalizes to another independent group of subjects that may exhibit subtle anatomical changes as observed in PSCs (Klöppel et al., 2009). To assess the robustness of the method against rather prominent anatomical variations, two subjects with enlarged CSF space were included. One subject (age = 79 years, male) showed significantly enlarged lateral ventricles. The other subject (age = 53 years, female) had a sub-cerebellar cyst. Written informed consent was obtained from all participants as supervised by the local Ethics committee.

### Data acquisition

All participants were scanned on a 3 T whole-body MRI scanner (Magnetom TIM Trio, Siemens Healthcare, Erlangen, Germany) operated with a 12-channel RF head receive coil and RF body transmit coil. Three 3D multi-echo FLASH datasets were acquired (as part of a multi-parameter mapping protocol; (Weiskopf and Helms, 2008)) with predominantly proton density weighting (repetition time TR = 23.7 ms, flip angle α = 6°), T1 weighting (TR/α = 18.7 ms/20°), and magnetization transfer weighting (MTw; TR/α = 23.7 ms/6°; excitation preceded by an off-resonance Gaussian MT pulse) and the following parameters: 1 mm isotropic resolution, 176 sagittal partitions, field of view (FOV) = 256 mm × 240 mm, matrix = 256 × 240 × 176, GRAPPA factor 2 in phase-encoding (PE) direction, 6/8 partial Fourier in partition direction, non-selective RF excitation, total acquisition time ~ 19 min (Weiskopf and Helms, 2008; Helms et al., 2009). The MTw data were not used in this study. The acquisition parameters of the FLASH sequences were optimized in a previous study (Weiskopf and Helms, 2008) for a trade-off between high SNR, small bias due to imperfect RF spoiling, < 20 min total scan time, low specific absorption rate (SAR), 1 mm isotropic resolution and whole-brain coverage. The use of multi-echo readouts with high readout bandwidths (BW = 425 Hz/pixel) reduced chemical shift artifacts and maintained a high SNR (Helms and Dechent, 2009).

Maps of the local B1^+^ field (ψ_m_) were measured and estimated from a 3D EPI acquisition of spin and stimulated echoes (SE and STE) with different refocusing flip angles (Lutti et al., 2010a). Imaging parameters were: matrix = 64 × 48 × 48, FOV = 256 mm × 192 mm × 192 mm (17% oversampling along the partition direction), TE_SE_ (echo time)/TE_STE_/TM (mixing time)/TR = 33.2/66.73/33.53/500 ms, acquisition time 2 min 20 s. The flip angles of the SE/STE refocusing pulses were varied between 160°/80° and 200°/100° in steps of 10°/5°.

To correct for off-resonance artifacts in the 3D EPI flip angle maps, an additional B_0_ map was acquired with the following parameters (Hutton et al., 2002; Lutti et al., 2010a): 64 axial slices, slice thickness = 2 mm, inter-slice gap = 1 mm, TR = 1020 ms, TE1/TE2 = 10/12.46 ms, α = 90°, FOV = 192 mm × 192 mm, matrix = 64 × 64, left-right PE direction, BW = 260 Hz/pixel, flow compensation, acquisition time ~ 2 min.

### Image processing

Data processing and analysis were performed with SPM8 (http://www.fil.ion.ucl.ac.uk/spm; (Friston et al., 2007)) and custom-made scripts in MATLAB 7.8 (The Mathworks, Natick, MA, USA).

Maps of the apparent R1 (R1_app_) were calculated from the T1w and PDw data according to Eq. (1) using the nominal flip angle value as entered on the scanner console. UNICORT was implemented using the “New Segment” toolbox in SPM8 and applied to estimate the bias field (η) and the corrected R1 maps (R1_UNICORT_) from the R1_app_ maps (Eq. (3)). Before estimating the bias field R1_app_ maps were masked with a mask of the head (including brain, skull and neck) determined from the low contrast PDw image using a histogram based threshold. The signal intensity of any voxel included in this mask had to exceed five times the modal score of the intensity distribution of the PDw image.

For validation of the UNICORT correction against the current established standard, R1_app_ maps were also corrected using measured B1^+^ maps (ψ_m_), yielding R1_m_ maps (Helms et al., 2008a; Preibisch and Deichmann, 2009; Lutti et al., 2010a). These R1_m_ maps were further corrected for bias due to imperfect spoiling of transverse coherences using the method reported by Preibisch and Deichmann (2009) and recalibrated for the FLASH sequence parameters used here. The deviation of UNICORT corrected R1 maps (D_UNICORT_) from the established standard was estimated by voxel-wise comparison with the B1^+^ map corrected R1 maps as follows:

(5) $$D_{\text{UNICORT}}=2\;\left|R1_{\text{UNICORT}}-R1_{\text{m}}\right|/\left(R1_{\text{UNICORT}}+\;R1_{\text{m}}\right)$$

As an aggregate measure, the median of D_UNICORT_ was determined across the whole brain (as defined by the GM, WM, CSF partitions and head mask). The analogous accuracy measure was calculated for the apparent R1_app_ maps, i.e.,

(6) $$D_{\text{app}}=2\left|R1_{\text{app}}-R1_{\text{m}}\right|/\left(R1_{\text{app}}+R1_{\text{m}}\right)$$

for comparison of D_UNICORT_ to the actual RF bias at 3 T,

To determine the optimal parameter settings for UNICORT, its performance was assessed for a range of regularization constants, κ = (10^− 5^, 10^− 4^, 10^− 3^, 10^− 2^, 10^− 1^), and smoothness constants of the bias field, FWHM = (30, 60, 90, 120, 150 mm), using the group of healthy volunteers. The parameters' influence was tested using a 5 × 5 factorial repeated measures ANOVA analysis implemented in SPSS Statistics 17.0 (SPSS Inc., Chicago, IL). The other settings of the “New Segment” toolbox were fixed at their default values (see Appendix A). All measures were calculated based on the images in native subject space.

In addition to the assessment of UNICORT performance for R1 correction, the modelling of the underlying B1^+^ inhomogeneities as determined by Eq. (4) was also assessed. Analogous to the deviation measure for R1 maps, a voxel-wise comparison of B1^+^ maps estimated by UNICORT (B1_UNICORT_) and measured using the 3D EPI method (B1_m_) was defined as:

(7) $$D_{B1}=2\;\left|B1_{\text{UNICORT}}-B1_{\text{m}}\right|/\left(B1_{\text{UNICORT}}+B1_{\text{m}}\right)$$

As an aggregate measure, the median of D_B1_ was determined for the whole brain.

For visualization and assessment of local correction effectiveness, R1_app_, R1_UNICORT_ and R1_m_ maps, their differential maps and brain masks were spatially normalized using the diffeomorphic image registration DARTEL (Ashburner, 2007), and smoothed by convolving with an isotropic Gaussian kernel (FWHM = 8 × 8 × 8 mm^3^). This smoothing accounted for the amounts of expansion and contraction incurred in the warping so that regional averages of R1 values were preserved as far as possible (Draganski et al., submitted for publication; Hutton et al., 2009). The smoothed and warped images were then averaged across the group and masked with a group brain mask (derived from averaged thresholded, individual masks and eroded/dilated).

The analyses were performed for the healthy volunteer and PSC groups separately. The two subjects with atypical brain anatomy underwent the same analysis except for the spatial normalization step because the results were assessed in native space. In order to assess the performance of UNICORT in the two different groups, a two-tailed two-sample t-test implemented in SPSS Statistics 17.0 was used to test for significant differences between D_UNICORT_ of the healthy volunteer and PSC group. For all statistical tests p < 0.05 was considered significant.

## Results

**Fig. 1:**
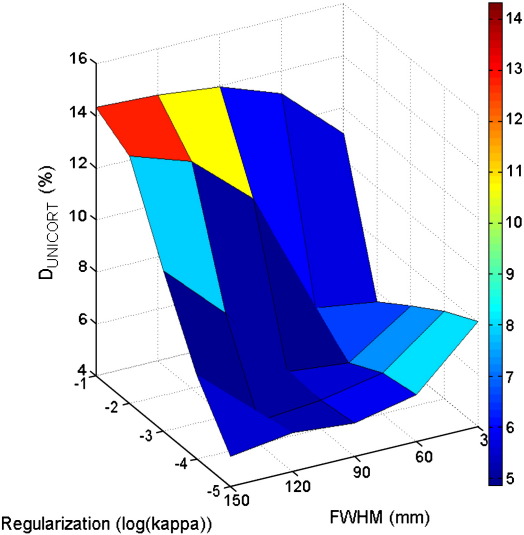
Dependence of UNICORT performance on the choice of the smoothness (FWHM) and regularization (κ) parameters (see also Table 1). Deviation of UNICORT corrected R1 maps (D_unicort_) from the established standard using measured B1^+^ maps.

UNICORT generally reduced the error in R1 (i.e., deviation from the established standard), but the choice of the smoothness (FWHM) and regularization (κ) parameters influenced the quality of R1_UNICORT_ maps significantly (Table 1 and Fig. 1). The ANOVA revealed a significant main effect of FWHM (F = 15.7, d.f. = 4) and κ (F = 36.4, d.f. = 4) and a significant interaction of κ × FWHM (F = 76.0, d.f. = 16). The smallest median percent deviation in the UNICORT corrected R1_UNICORT_ maps was D_UNICORT_ = 4.9%. It was found for two different parameter sets: 1) κ = 10^− 3^ and FWHM = 60 mm and 2) κ = 10^− 4^ and FWHM = 120 mm. For the further investigations we chose the first parameter set, since for this one small change in κ and FWHM leads to smaller changes in D_UNICORT_ (Table 1).

**Fig. 2:**
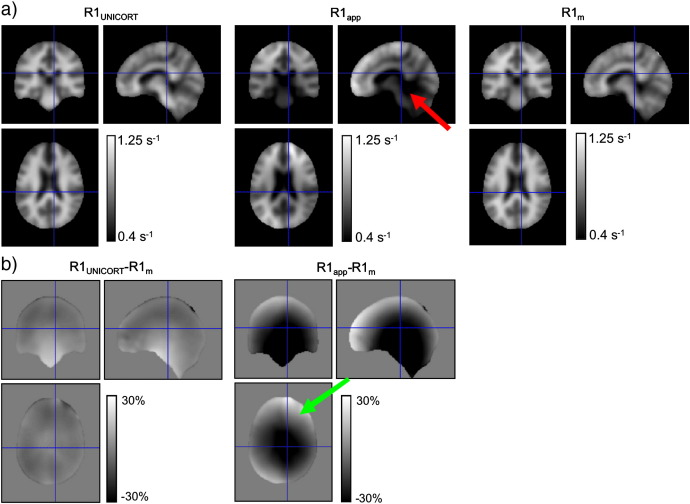
Spatially normalized and averaged R1 maps of healthy volunteers with different corrections for RF transmit field inhomogeneities. (a) R1 map corrected with UNICORT (R1_UNICORT_; left column), uncorrected apparent R1 map (R1_app_; center column), R1 map corrected with measured B1^+^ map (R1_m_; right column). In the uncorrected map, the spuriously increased R1 values in the center of the brain due to RF inhomogeneities can be well seen (red arrow). (b) Percent differences in R1 values between the B1^+^ map corrected R1 map (R1_m_) and the UNICORT (R1_UNICORT_; left column) or uncorrected apparent (R1_app_; right column) R1 map, respectively. In the difference between R1_app_ and R1_m_ the typical asymmetric RF inhomogeneities can be appreciated (green arrow).

With the optimal setting UNICORT significantly reduced the error in the R1 maps seen in central and marginal brain regions where the bias due to B1^+^ inhomogeneities was most apparent (see red arrow in Fig. 2a).

**Fig. 3:**
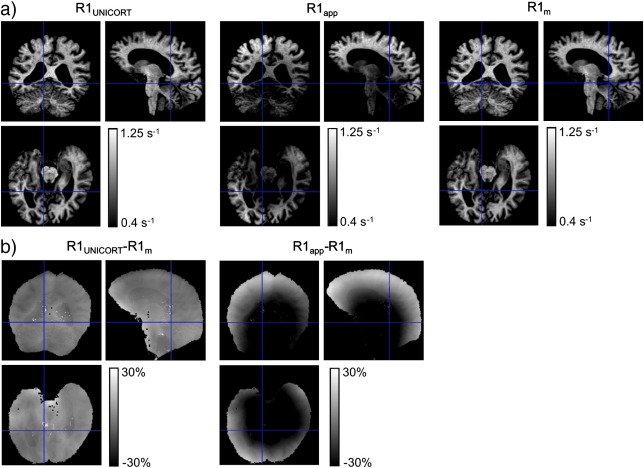
R1 maps of single subject with enlarged ventricles with different corrections for RF transmit field inhomogeneities. For detailed description, see Fig. 2. A similar pattern and magnitude of bias reduction was observed as in the group of healthy volunteers (Fig. 2).

UNICORT also removed the more subtle flip angle bias that is well described in the literature (Sled and Pike, 1998) and appeared as an asymmetric pattern in the R1_app_ maps (see green arrow in Fig. 2b). Even for the subject with enlarged ventricles, the bias in R1 maps was as effectively reduced as for the other groups (Fig. 3), indicating the robustness of the method.

Across the group of healthy volunteers, UNICORT reduced the median percent deviation in the R1 maps from D_app_ = 14.5% ± 0.7% (mean ± standard error) to D_UNICORT_ = 4.9% ± 0.6% (Table 1). In other words, it reduced the relative bias due to B1^+^ inhomogeneities by more than 66%. Similarly, in the PSC group the deviation was reduced from D_app_ = 14.4% ± 0.4% to D_UNICORT_ = 4.4% ± 0.2%. The minimal difference in D_UNICORT_ for the healthy volunteers and PSCs was not significant (t = 0.83, d.f. = 14, p > 0.41). In the subject with enlarged ventricles (Fig. 3) the deviation was reduced from D_app_ = 14.1% to D_UNICORT_ = 3.1%. Note that the fine anatomical detail is preserved on the UNICORT R1 maps (Fig. 3). In the subject with the sub-cerebellar cyst the deviation was reduced from D_app_ = 13.4% to D_UNICORT_ = 3.6%. To assess the residual error of UNICORT corrected R1 values, an independent further measure of R1 was estimated using an inversion recovery (IR) acquisition (see Appendix A). These results showed that the residual error of the UNICORT corrected R1 values was small (around 5%).

**Fig. 4:**
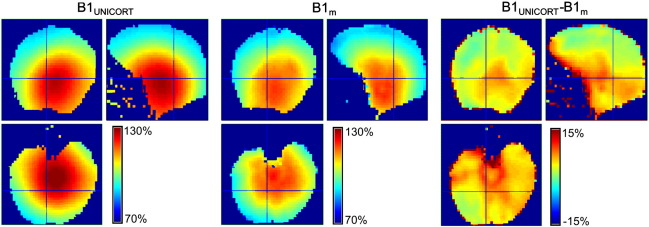
B1^+^ map (in percent of the nominal flip angle) of single subject (same as in Fig. 3) estimated by UNICORT (B1_UNICORT_; left column) and measured with the 3D EPI technique (B1_m_; center column). Percent differences in B1^+^ values between B1_UNICORT_ and B1_m_ (B1_UNICORT_ − B1_m_; right column). At the edge of the brain, in the connective tissue and muscles the measured B1^+^ maps are less accurate and cause edge artifacts.

UNICORT B1^+^ maps (Fig. 4) showed only small deviations from the measured B1^+^ maps with a mean D_B1_ = 4.2% ± 0.7% and D_B1_ = 3.9% ± 0.5% (mean ± standard error) for the healthy control and the PSC group respectively (Fig. 6). In the two subjects with atypical anatomy, UNICORT B1^+^ maps showed similar deviations from the measured B1^+^ maps with D_B1_ = 4.6% and D_B1_ = 4.2%, respectively.

**Fig. 5:**
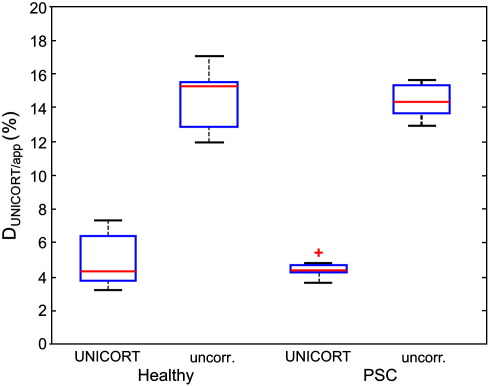
Median percent deviation of the UNICORT corrected and uncorrected R1_app_ maps compared to the B1^+^ map corrected R1_m_ maps. Descriptive statistics are provided for the group of healthy volunteers and presymptomatic Huntington disease gene mutation carriers (PSC): blue box = 25%/75% percentile, red line = median, black whisker = most extreme data value excluding outliers, red cross = outlier (probability < 0.01 under assumption of normally distributed data).

**Fig. 6:**
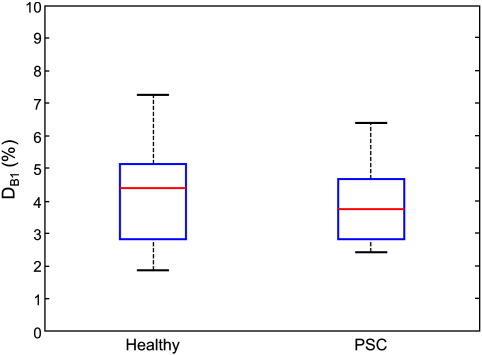
Median percent deviation of the B1^+^ map estimated by UNICORT from the measured B1^+^ map for the group of healthy volunteers and presymptomatic Huntington disease gene mutation carriers (PSC). For explanation of the box plot see Fig. 5.

Overall, UNICORT performed well without any indication for significant differences in performance between the two groups and the single cases (Figs. 5 and 6).

## Discussion

We developed and validated a post-processing approach for correcting bias in R1 maps (UNICORT) due to RF transmit field (B1^+^) inhomogeneities that uses a probabilistic framework for simultaneous image segmentation, registration and bias correction (Appendix A and Ashburner and Friston (2005)). The comparison with the established B1^+^ map based correction (Helms et al., 2008a; Lutti et al., 2010a) revealed that UNICORT reduces the error in R1 values by more than 66% with a residual mean error of less than 5%. The small residual error of UNICORT corrected R1 values was also confirmed by comparison with an independent inversion recovery R1 mapping technique (see Appendix B). UNICORT also reliably estimates the underlying B1^+^ inhomogeneities with a small mean error of approximately 4%.

Unlike conventional methods for correction of RF inhomogeneities, UNICORT does not require fast whole-brain B1^+^ mapping sequences which are usually not available on clinical scanners and are known to be difficult to implement (Lutti et al., 2010a). UNICORT can be easily implemented and applied, since the basic framework and implementation of the underlying unified segmentation are freely available via the SPM8 distribution (“New Segment” toolbox as described in Appendix A). The method is computationally efficient. Correction of a single dataset takes approximately 5 min on modern computer hardware (Intel Xeon W3570 3.2 GHz with 12 GB RAM; Intel Corp., Santa Clara, CA, USA).

The high performance of UNICORT can be attributed to the realistic physical model on which it is based. It exploits the fact that the modelled bias field is a smooth 3D field (Sled and Pike, 1998; Lutti et al., 2010a) and simply multiplicatively scales the R1 values obtained by the VFA R1 mapping method (Helms et al., 2008a; Dathe and Helms, 2010), as theoretically derived and experimentally validated. It also models the distribution of R1 values of GM, WM and CSF by a mixture of Gaussian distributions that do not vary across the brain. Even in the case of the slight variation of tissue specific T1 values in the brain this model is still applicable as empirically demonstrated for T1 at a range of static field strengths (Oros-Peusquens et al., 2008). Note that due to the relatively low noise and high mean values of the T1 distributions, this also holds for the distribution of R1 (= 1/T1) values.

While the implicit assumptions of UNICORT apply to any R1 map (obtained by whatever method, e.g., Look–Locker based methods (Zaitsev et al., 2003)), the observed relation between the multiplicative bias field and the inverse square of the B1^+^ field pertains specifically to the VFA R1 mapping method. In this case, UNICORT additionally provides a measure for the B1^+^ field heterogeneity through Eq. (4).

Compared to another method for joint RF inhomogeneity correction and segmentation proposed by Chen et al. (2009), UNICORT allows the bias field to vary in 3 dimensions in contrast to only the head–feet direction. This constitutes a more realistic model considering the patterns of variation of the B1^+^ field. In addition, we successfully validated our approach by extensive comparisons with the established standard R1 maps corrected by B1^+^ maps (Lutti et al., 2010a; Preibisch and Deichmann, 2009; Helms et al., 2008a).

### Error in UNICORT estimates

The 5% residual error in R1 values after UNICORT correction is approximately in the range of bias observed in other commonly used T1 mapping methods at 3 T. For example, Wright et al. (2008) observed mean differences of approximately 19% in GM and 4% in WM T1 values in a comparison of IR-TSE and MPRAGE based T1 mapping. Deoni (2007) reported a bias < 5% in the T1 value for a FLASH-based T1 mapping approach (DESPOT1-HIFI) compared to IR-SE measurements. The precision of dual angle 3D FLASH T1/R1 mapping is high due to the high duty cycle and 3D acquisition mode as shown previously (Deoni et al., 2003) and confirmed in this study with coefficients of variation < 9% despite the short acquisition time (< 12.5 min) and high 1 mm isotropic resolution (see Appendix B).

The very high accuracy of the R1 maps using optimized B1^+^ maps (see Appendix B) justifies the use of this method as the established standard for comparison. The remaining errors in UNICORT corrected R1 and estimated B1^+^ maps (compared to this standard) are likely to originate from different sources. The particular model of the bias field influences how well the actual physical transmit field inhomogeneities can be approximated. For example, our analysis of how the smoothness and regularization of the bias field affects UNICORT showed that only the optimal choice of parameters reduced the error in R1 maps by more than 66% and some suboptimal parameters lead to no reduction in error at all. There are also additional settings in the “New Segment” toolbox underlying UNICORT for which the impact was not investigated systematically, such as the regularization of the warp field, number of tissue classes or modelling of their intensity distributions (non-parametric or mixture of Gaussians). We also note that the settings of the inhomogeneity correction were optimized for a minimal aggregate error in UNICORT R1 maps, and that the quality of UNICORT B1^+^ maps may be improved by further optimization of parameters.

UNICORT assumes that the manufacturer's adjustment procedures set the RF transmit amplitude so that the mean flip angle across the whole brain equals the nominal flip angle entered on the console. The acquired B1^+^ maps support this assumption for our MRI scanner, since the mean deviation between the nominal flip angle and measured mean flip angle across the healthy volunteer and PSC groups was approximately 0.6% ± 1.3% (mean ± standard deviation across groups). Although the RF adjustment is theoretically a robust procedure and our experimental results support this assumption, a degree of inaccuracy can be expected, especially when working at higher field strengths or with different RF transmit coils.

Although we have used an optimized B1^+^ map based correction, the B1^+^ maps are less reliable in voxels containing CSF due to its long T1 times or in areas affected by physiological noise/susceptibility artifacts (Lutti et al., 2010a). Some errors in B1^+^ map corrected R1 values may also be attributed to the imperfect spoiling of the PDw/T1w FLASH acquisitions that used standard RF and gradient spoiling. Recent studies (Preibisch and Deichmann, 2009) have shown that residual coherent transverse magnetization may cause relatively small deviations (compared to B1^+^ inhomogeneities) from the Ernst equation. We employed additional post-processing in order to reduce the bias due to these higher order effects for the B1^+^ map based correction (Preibisch and Deichmann, 2009). Despite these optimizations it is conceivable that some of the differences between UNICORT and B1^+^ map corrected R1 maps actually originate from errors in the measured B1^+^ maps.

### General applicability

The consistent results obtained for the healthy volunteer and PSC groups indicate that UNICORT generalizes well to the population of subjects with normal or only minimally different brain anatomy. Even in the two subjects with atypically enlarged CSF spaces, the method reduced the bias in R1 values to a degree comparable with that of other cases. This suggests that UNICORT is widely applicable for accurate R1 mapping, probably even in cases of fairly atypical brain anatomy. However, it is clear that if the unified segmentation approach fails, the concomitant bias correction may be quite negatively affected. Therefore, caution should be exercised when using UNICORT on cases with severe anomalies, e.g., occurring in some cases of stroke or neurodegeneration (Gitelman et al., 2001). In particular, we recommend careful visual inspection of segmentation and UNICORT results.

We expect that UNICORT can be successfully adapted and applied to data from other MRI scanners and lower field strengths such as 1.5 T. Nevertheless, we recommend cross-validation with an established B1^+^ map based correction (Lutti et al., 2010a) or an alternative quantitative R1 mapping method (Tofts, 2003), since different RF transmitter adjustment procedures and RF transmit coils may lead to different results. In particular, RF transmitter adjustment procedures may vary considerably between scanners from different manufacturers, leading to different results and stability of the procedures.

Aside from the global offset in the B1^+^ field determined by the adjustment procedure, we expect the local relative B1^+^ inhomogeneities to be reasonably scanner independent and similar to the ones reported here as long as an RF transmit body coil is used. This can be assumed, since the B1^+^ inhomogeneities observed here are primarily determined by the electromagnetic tissue properties. We have studied a rather large group of 18 subjects in total and covered a wide age range from 23 to 65 years compared to previous methodological studies on T1 mapping, yielding a rather good approximation of the electromagnetic tissue properties of the wider population and the consequential B1^+^ inhomogeneities.

The tissue-induced B1^+^ inhomogeneities depend strongly on the strength of the static magnetic field. Lower field strengths and hence reduced levels of B1^+^ inhomogeneities (e.g., < 10% at 1.5 T) are not expected to reduce the effectiveness of UNICORT but the smoothness and regularization parameter should be optimized for this case. However at fields higher than 3 T, B1^+^ inhomogeneities can exceed 50% and exhibit higher spatial frequencies (Lutti et al., 2010b), perhaps requiring a different model for the bias field and not only the re-optimization of parameters.

UNICORT was validated on whole-brain high-resolution R1 maps. We do not recommend acquiring significantly smaller volumes or at significantly lower resolution without additional tests. A smaller brain coverage may not provide enough anatomical information for a reliable segmentation and the estimate of the average flip angle across the brain. Similarly, a lower spatial resolution will exacerbate partial volume effects and may impact on segmentation of the rather thin cortex (2–3 mm; Hutton et al., 2009).

We note that the unified segmentation approach correcting for a multiplicative bias has further potential applications in addition to R1 mapping. In general, it can be used to estimate and remove the signal bias from any type of data suffering from a multiplicative bias where intensity distributions from different tissue classes are sufficiently separated and the bias field is smooth. For example, it might be used for correction of the RF transmit and receive bias in PD mapping (Tofts, 2003).

## Conclusion

Quantitative R1 mapping using dual angle FLASH imaging with UNICORT RF transmit inhomogeneity correction significantly improves the accuracy of parameter estimates at 3 T compared to uncorrected maps. The results suggest that the method generalizes well to a wider population of subjects even with atypical brain anatomy. Unlike standard B1^+^ map based corrections that require specialized MR sequences, UNICORT is easy to implement and apply, since the fundamental probabilistic framework for unified segmentation and bias correction is implemented in SPM8 and publicly available. Also the 3D FLASH acquisition sequences used for the variable flip angle R1 mapping are commonly available on clinical scanners. We believe that UNICORT will help to make fast, high-resolution, whole-brain R1 mapping accessible to the wider neuroimaging community and open up new fields of research.

## Figures and Tables

**Table 1:** Accuracy of UNICORT for group of healthy volunteers.

D_unicort_ (%)	FWHM (mm)				
κ	30	60	90	120	150
10^− 5^	8.0 ± 1.3	5.8 ± 0.9	5.3 ± 0.9	5.5 ± 1.2	5.2 ± 0.6
10^− 4^	7.4 ± 1.1	5.6 ± 0.9	5.1 ± 0.9	4.9 ± 0.6	7.2 ± 0.4
10^− 3^	6.5 ± 0.9	4.9 ± 0.6	5.1 ± 0.4	8.0 ± 0.3	10.2 ± 0.3
10^− 2^	5.6 ± 0.6	5.9 ± 0.4	10.7 ± 0.5	12.7 ± 0.6	13.5 ± 0.6
10^− 1^	11.0 ± 0.4	13.1 ± 0.6	14.0 ± 0.7	14.2 ± 0.7	14.3 ± 0.7

Median percent deviation of UNICORT corrected R1 values from RF map corrected values (mean ± standard error) depending on smoothness (FWHM) and regularization (κ) of the modelled bias field. For comparison, D_app_ = 14.5 ± 0.7% for uncorrected apparent R1_app_ maps.

## Appendix A Extensions in SPM 8 “New Segmentation”

The “New Segmentation” model of SPM8 (for detailed information on SPM8 release, see http://www.fil.ion.ucl.ac.uk/spm) is an extended version of the “Unified Segmentation” model (Ashburner and Friston, 2005). In principle, it may be applied to multi-spectral images, but this brief description will be limited to the case of segmenting a single image. For an image (e.g., the original R1 image) containing *I* voxels, where the intensity of each voxel is *y*_*i*_, the objective function minimized when fitting the model is:

(A1) $$E=-\sum_{i=1}^{I}\log\left(\sum_{k=1}^{K}b_{\mathrm{ik}}\left(\alpha \right)p\left(\frac{y_{i}}{\eta_{i}\left(\beta \right)}|\theta_{k}\right)\right)+\sum_{i=1}^{I}\log\left(\eta_{i}\left(\beta \right)\right)+\frac{1}{2}\beta^{T}C_{\beta }^{-1}\beta +\frac{1}{2}\alpha^{T}C_{\alpha }^{-1}\alpha$$

Segmentation is assisted by overlaying deformed tissue probability maps (TPMs) on to the data, where there are typically *K* = 6 different tissues within the model. The TPMs (of GM, WM, CSF, bone, soft tissue and air) have been pre-computed, but the deformation required to align them is estimated by the algorithm. A small deformation model is used (after an initial affine alignment), which is parameterized by the vector *α*. The estimated probability of obtaining the *k*th tissue class at voxel *i* is denoted by *b*_*ik*_(*α*).

Typically, *α* contains about 1,500,000 elements, which allow reasonably detailed displacement fields to be modelled. To prevent over-fitting, a regularization term penalises the bending energy of the displacements. The precision matrix, *C*_*α*_^− 1^, encodes this energy term, such that the amount of bending is computed from $\frac{1}{2}\alpha^{T}C_{\alpha }^{-1}\alpha$.

A single user specified hyper-parameter determines the weighting of the regularization. The default value for this setting is 4, which was selected to give reasonable results when modelling T1w images. When estimating the various model parameters, not all of the voxels within the image are used. To save time, the image is sampled approximately every 3 mm (the default setting).

A component is included for estimating the signal inhomogeneity of the image data, such that the value of the field at voxel *i* is given by *η*_*i*_(*β*). The field is parameterized by a vector of *N*_*β*_ parameters (*β*), and generated by taking the exponential of a linear combination of 3D cosine transform spatial basis functions (A)

(A2) $$\eta_{i}\left(\beta \right)=\exp\left(-\sum_{n=1}^{N_{\beta }}\alpha_{\mathrm{in}}\beta_{n}\right)$$

As in the case of the displacements, there is a regularization term penalising large deviations away from a uniform inhomogeneity field. This regularization term is encoded by the precision matrix *C*_*β*_^− 1^, and is specified using prior knowledge of the range of intensity nonuniformity likely to be encountered in the data.

Segmentation is driven by the intensities within the image. Assigning tissue class memberships to voxels requires the intensity distribution of each of the *K* tissue types to be known. Intensity distributions may be parameterized in a number of ways, and two alternative forms are available within the SPM8 implementation. The first approach is to parameterize the probability density of image intensities within the *k*th class using a mixture of Gaussians (used for UNICORT). Here *θ*_*k*_ is used to represent the *L*_*k*_ mixing proportions (*γ*), means (*μ*) and variances (*σ*^2^). By default, intensities within the six tissue classes are modelled using a mixture of two Gaussians to represent GM, two for WM, two for CSF, three for bone, four for soft tissue (other than brain) and two for air/background.

(A3) $$p\left(y|\theta_{k}\right)=\sum_{l=1}^{L_{k}}\frac{\gamma_{l}}{\sqrt{2\pi \sigma_{l}^{2}}}\exp\left(-\frac{1}{2\sigma_{l}^{2}}{\left(y-\mu_{l}\right)}^{2}\right)$$

The other parameterization approach (not used within the present work) uses a histogram to represent the probability densities. The histogram approach avoids some of the local optima involved when fitting a mixture of Gaussians, and additional regularization terms are included to penalise the roughness of the histograms. Unfortunately though, the use of histograms does not generalize easily to multi-spectral data.

The actual segmentation is preceded by an affine registration with the TPM data. This uses the non-parametric framework to define the objective function, but without any inhomogeneity correction. Shears and zooms of the estimated affine transform are penalized so that they conform to the range of values that would be expected when aligning the TPMs with the brains of European subjects (the default setting).

## Appendix B Accuracy and precision

Two additional experiments assessed the accuracy and precision of the UNICORT corrected R1 maps beyond the internal comparison with R1 maps corrected using measured B1^+^ maps. Two participants (female, age = 25/40 years) who were not part of the groups studied in the main experiment were recruited for these experiments. Written informed consent was obtained as supervised by the local Ethics committee. Both participants were scanned with the same UNICORT R1 mapping protocol used in the main experiment (see Methods).

### B.1 Accuracy

To determine a measure of R1 independent from the dual angle FLASH acquisition, one participant was scanned with an inversion recovery turbo spin echo (IR-TSE) sequence at different inversion times (TI = 3000/2000/1350/950/650/450/300/200 ms). Although IR-TSE is often regarded a gold standard in R1 mapping, it is not an alternative to the FLASH-based R1 mapping due to prohibitively long acquisition times when high resolution and/or coverage is required (Tofts, 2003).

A single axial slice through the caudate nucleus, lateral ventricles and corpus callosum was acquired with the following parameters: FOV = 240 mm × 180 mm, matrix = 192 × 144, resolution = 1.25 mm × 1.25 mm × 5 mm, slice thickness = 5 mm, turbo factor = 7, left-right PE direction, TE = 12 ms, TR = TI + 3000 ms, refocusing pulse flip angle = 150°, BW = 130 Hz/pixel.

The R1 value in each voxel was determined by non-linearly fitting the measured signal *S*(*TI*) to the IR signal equation (Nelder–Mead minimization as implemented in MATLAB 7.8): *S*(*TI*) = *S*_0_(1 − *ρ* exp(− *TI* ⋅ *R*1)). *S*0 is the maximal signal at full relaxation and *ρ* reflects the inversion efficiency.

Accuracy was determined within GM and WM (as determined by the automatic segmentation step and thresholded at 99% probability) and within two ROIs manually drawn in the caudate nuclei and genu of the corpus callosum on the IR-TSE image using MRIcroN (http://www.cabiatl.com/mricro/). The mean and standard deviation (sd) of R1 values in each ROI were determined for the different R1 maps: IR-TSE based (R1_IR_), dual angle FLASH R1 maps with B1^+^ map correction (R1_m_), UNICORT correction (R1_UNICORT_) or no correction (R1_app_; after resampling to the resolution of the IR-TSE scan).

The R1 values in GM were: R1_IR_ = 0.688 ± 0.055 s^− 1^, R1_m_ = 0.646 ± 0.045 s^− 1^, R1_UNICORT_ = 0.619 ± 0.048 s^− 1^, R1_app_ = 0.571 ± 0.072 s^− 1^ (mean ± sd). In the WM mask the R1 values were: R1_IR_ = 1.065 ± 0.081 s^− 1^, R1_m_ = 1.084 ± 0.068 s^− 1^, R1_UNICORT_ = 1.054 ± 0.058 s^− 1^, R1_app_ = 0.959 ± 0.163 s^− 1^.

The R1 values in the caudate nuclei were: R1_IR_ = 0.705 ± 0.023 s^− 1^, R1_m_ = 0.683 ± 0.020 s^− 1^, R1_UNICORT_ = 0.647 ± 0.024 s^− 1^, R1_app_ = 0.540 ± 0.024 s^− 1^. The R1 values in the genu of the corpus callosum were: R1_IR_ = 1.182 ± 0.050 s^− 1^, R1_m_ = 1.188 ± 0.037 s^− 1^, R1_UNICORT_ = 1.123 ± 0.38 s^− 1^, R1_app_ = 1.035 ± 0.032 s^− 1^.

UNICORT reduced the deviation from the IR-TSE R1 map significantly from minimally 10% and maximally 23% down to 1% and 8%, respectively. The residual deviation of UNICORT R1 values from the IR-TSE values (maximal 10% in GM) was only slightly larger than previously reported values for other R1/T1 mapping approaches (e.g., < 5% Deoni (2007), ~ 4–19% for Wright et al. (2008), ~ 3–13% for Preibisch and Deichmann (2009) at 3 T; ~ 7% for Deoni et al. (2003) at 1.5 T).

The accuracy of the R1 maps corrected with the measured B1^+^ map was very high with only 0.5–3.2% deviation. Only the deviation in GM exceeded 6%, but may be due to the rather low resolution of the IR-TSE based R1 map for mapping of the cortical GM. These small deviations confirm the B1^+^ map corrected R1 maps as the established standard for validating the UNICORT correction approach. It should be noted that the comparisons between single slice IR-TSE R1 maps and whole-brain R1 maps with different resolutions allow limited conclusions only. Therefore, the group analyses on the UNICORT performance was conducted based on the same high-resolution whole-brain R1 maps with different corrections.

### B.2 Precision

As a measure of the precision the scan–rescan variance was used, since it is not biased by different image reconstruction methods (e.g., GRAPPA) and includes the most relevant variance components. Thus, one participant was scanned twice in a single session, resulting in two UNICORT R1 maps. The standard deviation of the voxel-by-voxel-wise difference of the two maps was determined within GM and WM (as determined by the automatic segmentation step and thresholded at 99% probability). The standard deviation in GM and WM was 0.064 s^− 1^ and 0.077 s^− 1^, respectively. The mean R1 in GM and WM was 0.730 s^− 1^ and 1.173 s^− 1^ (T1 = 1370/852 ms). The corresponding coefficient of variation (CV) was 6.5% and 8.7%, thus similar to published results of approximately 5% (e.g., Deoni, 2007).
